# Gestational hypoxia modulates expression of corticotropin‐releasing hormone and arginine vasopressin in the paraventricular nucleus in the ovine fetus

**DOI:** 10.14814/phy2.12643

**Published:** 2016-01-05

**Authors:** Dean A. Myers, Krista Singleton, Christy Kenkel, Kanchan M. Kaushal, Charles A. Ducsay

**Affiliations:** ^1^ Department of Obstetrics and GynecologyUniversity of Oklahoma Health Sciences CenterOklahoma CityOklahoma; ^2^ School of MedicineThe Center for Perinatal BiologyLoma Linda UniversityLoma LindaCalifornia

**Keywords:** AVP, CRH, fetus, hypoxia, PVN, sheep

## Abstract

Maturation of the fetal hypothalamo–pituitary–adrenocortical (HPA) axis is critical for organ maturation necessary for the fetus to transition to the ex‐utero environment. Intrauterine stressors can hasten maturation of the HPA axis leading to fetal growth restriction and in sheep, premature birth. We have previously reported that high‐altitude mediated, long‐term‐moderate gestational hypoxia (LTH) during gestation has a significant impact on the fetal HPA axis. Significant effects were observed at the level of both the anterior pituitary and adrenal cortex resulting in elevated plasma ACTH during late gestation with decreased adrenocortical expression of enzymes rate limiting for cortisol synthesis. As such, these fetuses exhibited the normal ontogenic rise in fetal plasma cortisol but an exaggerated cortisol response to acute stress. This study extended these findings to ACTH secretagogue expression in the PVN using in situ hybridization. We report that the expression of AVP but not CRH was increased in the medial parvocellular PVN (mpPVN) in the LTH fetus. This represented an increase in both AVP mRNA per neuron as well as an increase in AVP hybridizing neurons with no increase in mpPVN CRH neurons. LTH had no effect on PVN volume, area of CRH or AVP hybridization, thus LTH did not have a trophic effect on the size of the nucleus. In conclusion, there appears to be a switch from CRH to AVP as a primary ACTH secretagogue in response to LTH, supporting our previous findings of increased anterior pituitary sensitivity to AVP over CRH in the LTH fetus.

## Introduction

The hypothalamo–pituitary–adrenal (HPA) axis of the fetus undergoes a functional maturation during the final weeks of gestation. This results in an exponential rise in fetal plasma cortisol that matures various organ systems (e.g., lung, liver, GI tract) fundamental for survival post‐birth. During this maturational process, there is increased expression of the major ACTH secretagogue, corticotropin‐releasing hormone (CRH) in the hypothalamic paraventricular nucleus (PVN) as well as increased expression of the ACTH precursor, proopiomelanocortin (POMC) in the anterior pituitary (Matthews and Challis 1995a, 1997; Myers and Myers 2000). In addition, there is a noted increase in the processing of POMC to ACTH in anterior pituitary corticotrophs that translates to an enhanced bioactivity of fetal plasma immunoreactive ACTH and mature, bioactive ACTH_1–39_ (Castro et al. 1993; Carr et al. 1995; Zehnder et al. 1995; Fora et al. 1996; Bell et al. 1998). These maturational changes in the PVN and anterior pituitary are accompanied by the increased adrenocortical expression of steroidogenic enzymes resulting in the increased cortisol synthesis. Stereotaxic lesion of the ovine fetal PVN during late gestation blocks the processing of POMC to ACTH in the fetal anterior pituitary. This inhibition results in a loss of circulating ACTH_1–39_ which in turn prevents the re‐emergence of the expression of the two key rate‐limiting enzymes necessary for cortisol synthesis (CYP11A1 and CYP17) and thus the late gestation surge in fetal plasma cortisol (McDonald et al. 1993; Bell et al. 1997, 2005). In addition to the late gestation surge in fetal plasma cortisol, the fetal HPA axis also provides the late gestational fetus with an essential defense mechanism; the ability to produce cortisol acutely, and thus the ability to maintain homeostasis in the face of potential physiologic stressors.

Hypoxia is a major common threat to the developing fetus and can occur at any point during gestation, or in some situations such as placental insufficiency, smoking, or high altitude, throughout gestation. Fetal hypoxia also varies in terms of both severity and duration with the fetus experiencing both acute (e.g., minutes to hours), sustained (hours to days), or long‐term (weeks to months) hypoxia. We have developed a model of high altitude (3820 m), moderate (pO_2_ ~80% of normoxic fetuses), long‐term hypoxia (LTH) in sheep where hypoxia is initiated at approximately 40 days gestation (dG; term ~146 dG) (Harvey et al. 1993; Adachi et al. 2004; Imamura et al. 2004; Ducsay et al. 2007b). We have reported a remarkable adaptation of the fetal HPA axis at the level of the anterior pituitary and adrenal cortex to LTH that enables the ovine fetus to undergo normal somatic growth by limiting the anticipated stimulatory effect of the LTH stressor and thus preserves the normal ontogenic rise in fetal plasma cortisol in late gestation (Myers and Ducsay 2012, 2014). These fetuses exhibit a seeming dipartite response to LTH. As anticipated, the LTH fetus has elevated basal plasma ACTH_1–39_ driven by enhanced anterior pituitary processing of POMC to ACTH_1–39_ (Myers et al. 2005). However, the adrenal cortex of the LTH fetus has equally adapted to limit the response to the elevated plasma ACTH_1–39_ with decreased expression of CYP11A1 and CYP17 and subsequent maintenance of normal basal cortisol concentrations. Paradoxically, the LTH fetuses nonetheless have increased cortisol production in response to an acute secondary stressor, mediated in part via enhanced ACTH_1–39_ release in response to the acute stress and through intra‐adrenal mechanisms involving nitric oxide (NO) (Monau et al. 2009, 2010; Ducsay and Myers 2011).

At the level of the anterior pituitary, we previously demonstrated that the LTH fetuses exhibit enhanced ACTH_1–39_ release to exogenous AVP, whereas the ACTH_1–39_ response to exogenous CRH remains similar to control fetuses (Ducsay et al. 2009). Expression of the AVP V1b receptor in the anterior pituitary is increased in the LTH fetuses, whereas the CRH type 1 receptor (CRH_1_) is decreased suggesting that these fetuses have adapted differential ACTH secretagogue regulation of ACTH production and release compared to control fetuses. However, we have not examined expression of CRH or AVP at the level of the parvocellular hypothalamic PVN to determine if LTH modifies expression of these two major ACTH‐releasing factors, providing yet another level of adaptation to this chronic moderate stressor. Thus, this study was conducted to examine the expression of CRH and AVP in the PVN of LTH fetal sheep using in situ hybridization. We hypothesized that LTH would up‐regulate the expression of CRH and AVP in the parvocellular PVN of the late gestation ovine fetus.

## Methods

### Animals

All procedures were conducted with the approval of the Institutional Animal Care and Use Committee (Loma Linda University School of Medicine). Pregnant ewes were maintained at 3820 m at the Barcroft Laboratory White Mountain Research Station from approximately 30 dG until 137–138 dG. Immediately after arrival at Loma Linda University Medical Center Animal Research Facility (elevation: 346 m), ewes were implanted with an arterial and nonocclusive tracheal catheter, as previously described (Adachi et al. 2004b; Ducsay et al. 2006, 2007a). Maternal PO_2_ for LTH animals was maintained at ~60 mmHg by adjusting humidified nitrogen flow through the maternal tracheal catheter. Normoxic control ewes were maintained near sea level (~300 m) throughout gestation. Between days 139 and 141 days of gestation, both control and LTH ewes were sedated with pentobarbital, intubated, and maintained under general anesthesia with 1.5–2% halothane in oxygen. Fetuses were then delivered through a midline laparotomy and immediately euthanized by exsanguination. Fetal brains were rapidly removed and hypothalami were dissected in accordance with the following anatomical features: immediately anterior (3 mm) to the optic chiasm, posterior to the mammillary bodies, and 5 mm either side of the midline. The floor of the lateral ventricles was used as the dorsal boundary (Myers et al. 1993). Hypothalami were rapidly frozen by immersion in liquid nitrogen‐cooled isopentane, covered in O.C.T. compound to prevent desiccation, and stored at −80°C until cryosectioned.

### In situ hybridization

The procedure for in situ hybridization detection and quantification of CRH and AVP mRNA was previously published for both fetal sheep and adult rat hypothalami in our laboratory (Myers et al. 1993; Shepard et al. 2003, 2006). Hypothalami were sectioned in the coronal plane (30 *μ*m thickness per section) on a cryostat; all sections were obtained through the entire PVN (~2–3000 *μ*m rostro‐caudal extent; determined by Nissl staining) and rapidly thaw mounted onto sialated slides (Superfrost Plus, Fisher Scientific) and stored at −80°C until hybridization. A one in five series of sections through the PVN were collected from each animal and one series of slides for each neuropeptide (CRH and AVP) was subjected to in situ hybridization per hypothalami. This procedure ensured complete representation of the nucleus.

At the time of hybridization, sections were equilibrated (10 min) at room temperature, and then fixed (5 min) in freshly prepared 4% paraformaldehyde (pH 6.8). Slides were washed twice in sodium and potassium phosphate‐0.9% sodium chloride (6.5 mmol/L; pH 7.2) and placed in freshly prepared 0.25% acetic anhydride in 0.1 mol/L triethanolamine (pH 8) for two successive 5‐min acetylations. Sections were then dehydrated and delipidiated by successive washes through ethanol and chloroform (1 min, 70% ethanol; 1 min, 85% ethanol; 2 min, 95% ethanol; 1 min, 100% ethanol; 5 min, chloroform; 1 min, 100% ethanol; 1 min, 95% ethanol) and air‐dried. All sections for this study were processed together for this and all subsequent steps.

Prehybridization was carried out by placing 100 *μ*L prehybridization solution [4 × SSC (1 × SSC = 0.15 mol/L sodium chloride; 0.015 mol/L sodium citrate; pH 7.2), 40% deionized formamide, 10% (wt/vol) dextran sulfate, denatured sonicated salmon sperm DNA (500 *μ*g/mL), tRNA (250 *μ*g/mL), 2.5 × Denhardt's (1 × Denhardt's = 1% solution of BSA, Ficoll, and oligonucleotide probes (polyvinylpyrrolidone), 4 μmol/L EDTA, 0.1% pyrophosphate, and 50 μmol/L α‐thio‐deoxy‐ATP] on each section. Sections were coverslipped with parafilm, and prehybridization was performed at 37°C for 2 h in a humidified chamber. Prehybridization solution was then removed, and fresh prehybridization solution (100 *μ*L) containing 10 μmol/L dithiothreitol (DTT) and 1 × l0^7^ cpm/mL ^35^S‐radiolabeled CRH or AVP probes was placed over each section, coverslipped with parafilm, and allowed to hybridize at 37°C overnight (~20 h). For CRH, we used two oligonucleotide probes (40‐mers) with similar G/C content, antisense to nucleotides 442–482, and 483–523 of ovine CRH cDNA (NCBI E00212.1). For AVP, two oligonucleotide probes (40‐mers) representing the unique glycoprotein domain of the AVP coding sequence (NCBI: NM_0011263341.1) not found in the highly conserved oxytocin gene (NCBI: X55131.1) were used. Oligonucleotides were labeled with terminal deoxynucleotidyl transferase with *α* ^35^S d‐ATP (SA. 1000–1500 Ci/mmol: New England Nuclear‐DuPont, Boston, MA) to a specific activity of 4620 Ci/mmol DNA. After hybridization, sections were briefly washed twice in 1 × SSC at 23°C, twice (30 min each time) in 1 × SSC at 55°C, and once (30 min) in 1 × SSC containing 0.1% Triton X‐100 at 23°C; briefly rinsed in water; then briefly immersed in 70% ethanol and air dried overnight. As previously published (Myers et al. 1993), the specificity of mRNA hybridization was determined by the ability of RNase‐A (10 *μ*g/mL) and RNase‐Tl (1 pg/mL) pretreatment to eliminate hybridization signal in sections containing PVN.

Sections were opposed to Hyperfilm‐ßmax film (Amersham, Arlington Heights, IL) at −20°C for 10 days for CRH and 7 days for AVP, developed (GBX developer, Eastman Kodak, Rochester, NY) and fixed (GBX fixer, Eastman Kodak) to verify specific labeling. Slides containing PVN were then dipped in nuclear emulsion (NBT2, Eastman Kodak) stored at 4°C for 45 days (CRH) or 28 days (AVP), developed (D19, Eastman Kodak) for five min at 16°C, fixed (Kodak fixer) and counterstained with 0.1% toluidine blue.

### Image analysis

The procedure for quantification of in situ hybridization has been described in detail for our laboratory (Shepard et al. 2003, 2006; Shepard and Myers 2008). Bright‐field images were collected using an Olympus BH‐40 microscope equipped with Olympus DP‐11 digital camera (1712 × 1368 pixel resolution). Image analysis was performed using NIH Image J (W. Rasband: http://imagej.nih.gov/ij/). Background counter staining (toluidine blue) was reduced in each image using Wratten 4A (Kodak) filtered light during image capture (Shepard et al. 2003, 2006; Shepard and Myers 2008). Once captured, images were subjected to high pass filtration to increase contrast between silver grains and underlying stained neurons. Images were then duplicated and the duplicated images superimposed on the originals. Maximum gray level of the histological staining was determined and subtracted from the duplicated images. Thus, on the duplicated images, only gray level for silver grains remained (background grayscale = 0; silver grain grayscale > 0). Stained neurons or small groups of neurons were outlined in the original image and the neuronal outlines transferred to the duplicate images allowing determination of silver grain number per unit area of PVN. The gray level for pixels over each outlined area was determined and the number of pixels greater than zero (representing the area of silver grains) was divided by the number of pixels per silver grain providing an accurate determination of silver grains per area (Shepard et al. 2003, 2006). In our prior published studies using this method, we reported a close linear relationship between manual counting of grains and this method with *r *>* *0.90 (Shepard et al. 2003, 2006). Background hybridization was calculated from 25 neurons outside of the PVN of each section. Specific hybridization was determined as an area for which the number of overlying silver grains was greater than two standard deviations above the background mean number of silver grains. Background number of silver grains was subtracted from hybridization signal of specifically labeled neurons.

**Figure 1:**
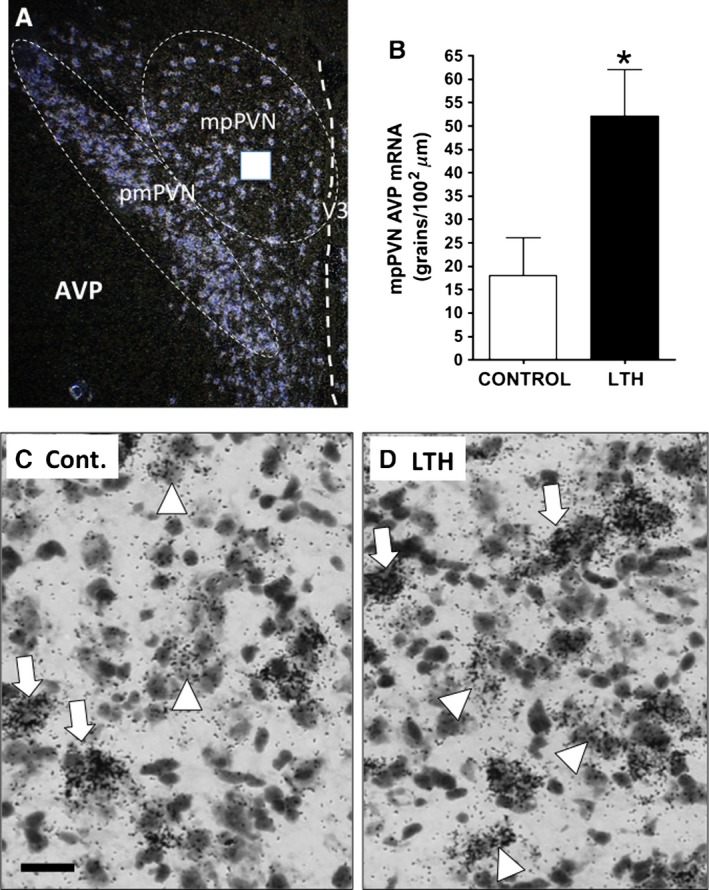
(A) Dark‐field image (2× objective magnification) of AVP hybridization in the mid‐region of the ovine fetal PVN with the posterior magnocellular (pmPVN) and medial parvocellular (mpPVN) divisions outlined. The white box represents the level of the mpPVN where images (C, D) were obtained (V3: third ventricle). (B) AVP mRNA levels in the mpPVN of control and LTH fetal sheep (*n* = 5/group; mean ± SEM). Medial parvocellular AVP mRNA was significantly elevated in the LTH fetuses. (C, D) Messenger RNA hybridization signal (silver grains) in the medial parvocellular PVN (mpPVN) of normoxic control (C) and LTH (D) fetal sheep (40× objective magnification, bar = 25 *μ*m). Arrows designate magnocellular AVP expressing neurons while arrowheads designate lower hybridization signal strength parvocellular AVP neurons.

For AVP mRNA within medial parvocellular neurons within the PVN, an imaging method was developed that allowed us to distinguish between magnocellular and parvocellular neurons in the PVN. In addition to their distinct anatomical location within the PVN (the lateral magnocellular division), scattered magnocellular neurons reside within the medial parvocellular (mpPVN) division of the PVN (Fig. 1). The presence of these neurons within the mpPVN excludes a general imaging of the entire mpPVN since magnocellular neurons in the PVN express neuropeptides at orders of magnitude greater than parvocellular neurons. As we previously published (Shepard et al. 2003), we identified magnocellular neurons by plotting the number of silver grains per unit area within neurons revealing a bimodal distribution of labeled neurons. Since magnocellular neurons express high levels of AVP, the distribution representing the most heavily labeled neurons were considered to be magnocellular and excluded from the data set (within the mpPVN these neurons had silver grains for AVP similar to those within the lateral magnocellular division per se). As predicted from previous descriptions, the majority of the heavily labeled AVP neurons resided within the magnocellular subdivision of the PVN with a few scattered magnocellular neurons located within the parvocellular subdivisions (Shepard et al. 2003).

Magnocellular AVP in the PVN and SON was quantified from the Hyperfilm‐ßmax film images since the silver grain development in the magnocellular neurons had reached the near saturation, nonlinear response level thus precluding the imaging from emulsion coated sections for these neurons. Thus, as previously described for our laboratory (Greenwood‐Van Meerveld et al. 2006), we quantified magnocellular AVP mRNA from film images in which silver grain development (at 7 days) was optimal for magnocellular signal strength with little grain development over the mpPVN region. Film images were obtained at 10× objective magnification, the region corresponding to the posterior magnocellular division of the PVN (pmPVN) and SON was outlined and gray scale determined using NIH Image J. The pmPVN and SON was imaged from at three consecutive sections for each fetus, bilaterally, and date for the six images averaged for each fetus.

For determination of total volume of the PVN, bright‐field images (2× objective magnification) were obtained throughout the rostral‐caudal extent of each PVN. Using NIH ImageJ 1.48 (running on Java 1.6), the boundaries of the PVN were traced and copied for each section containing PVN, typically approx. 10 sections representing ~2000 *μ*m rostral‐caudal for each fetus. The paired PVN were individually captured (traced) for each PVN, then the tracings were transferred to a new image and a stack was created for each unilateral PVN. Using ImageJ, a 3D image of each PVN was created and volume rendering applied. The volume was then calculated using ImageJ volume setting in the 3D measurements plugin. The volume is expressed in mm^3^. For the final analysis the volumes of the paired PVN are used (i.e., left and right side of the third ventricle). For the volume of the CRH (parvocellular PVN) and AVP (magno and parvocellular) hybridizing areas of the PVN, dark‐field images of silver grain hybridizing area were collected as above throughout the rostral‐caudal extent of the nucleus and volume determined as described above.

## Results

**Figure 2:**
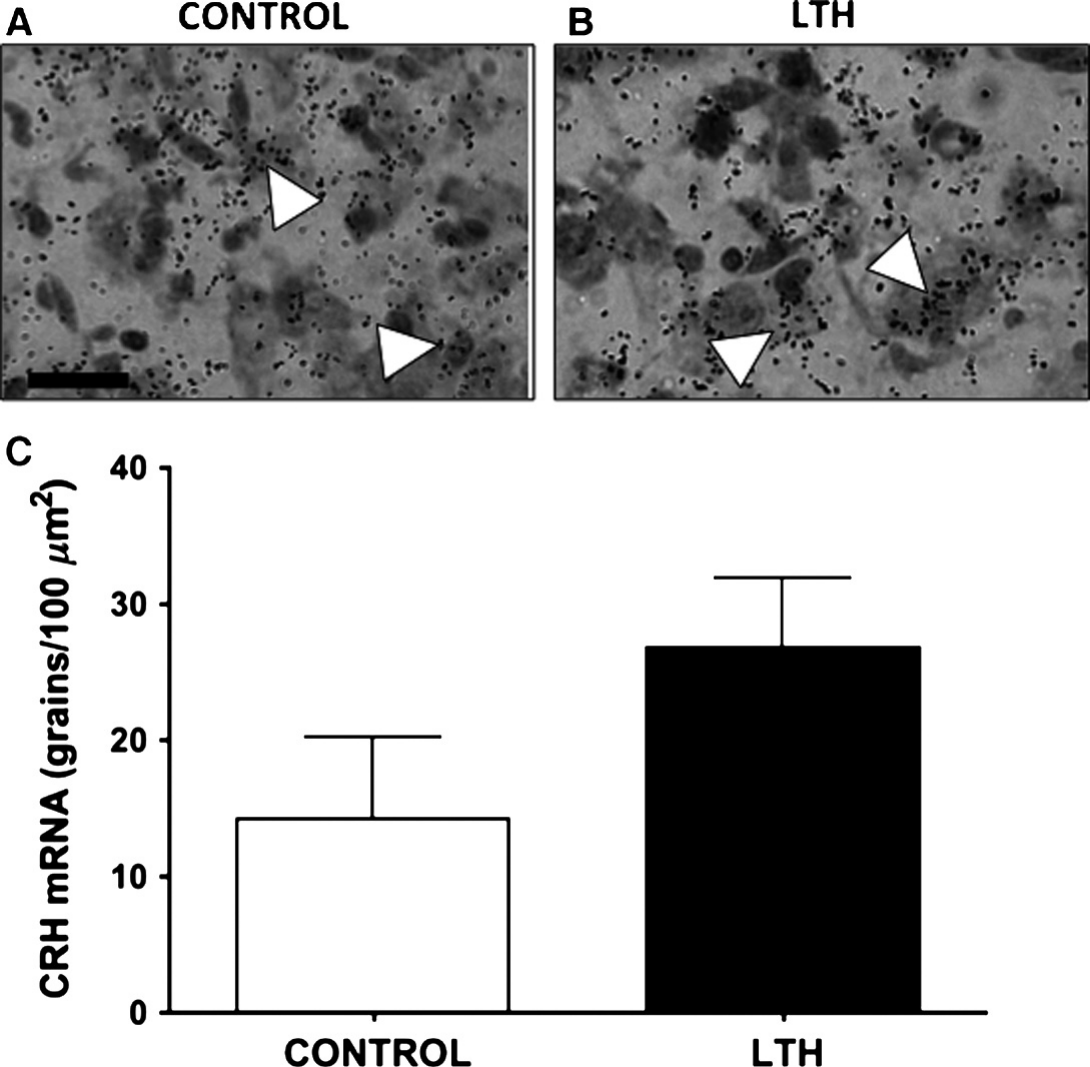
CRH mRNA hybridization signal (silver grains) in the medial parvocellular PVN (mpPVN) of normoxic control (A) and LTH (B) fetal sheep (40× objective magnification, bar = 25 *μ*m). (C) CRH mRNA levels in the mpPVN of Control and LTH fetal sheep (*n* = 5/group; mean ± SEM). There was a trend (0.07) for the CRH hybridization signal being elevated in the LTH mpPVN.

**Figure 3:**
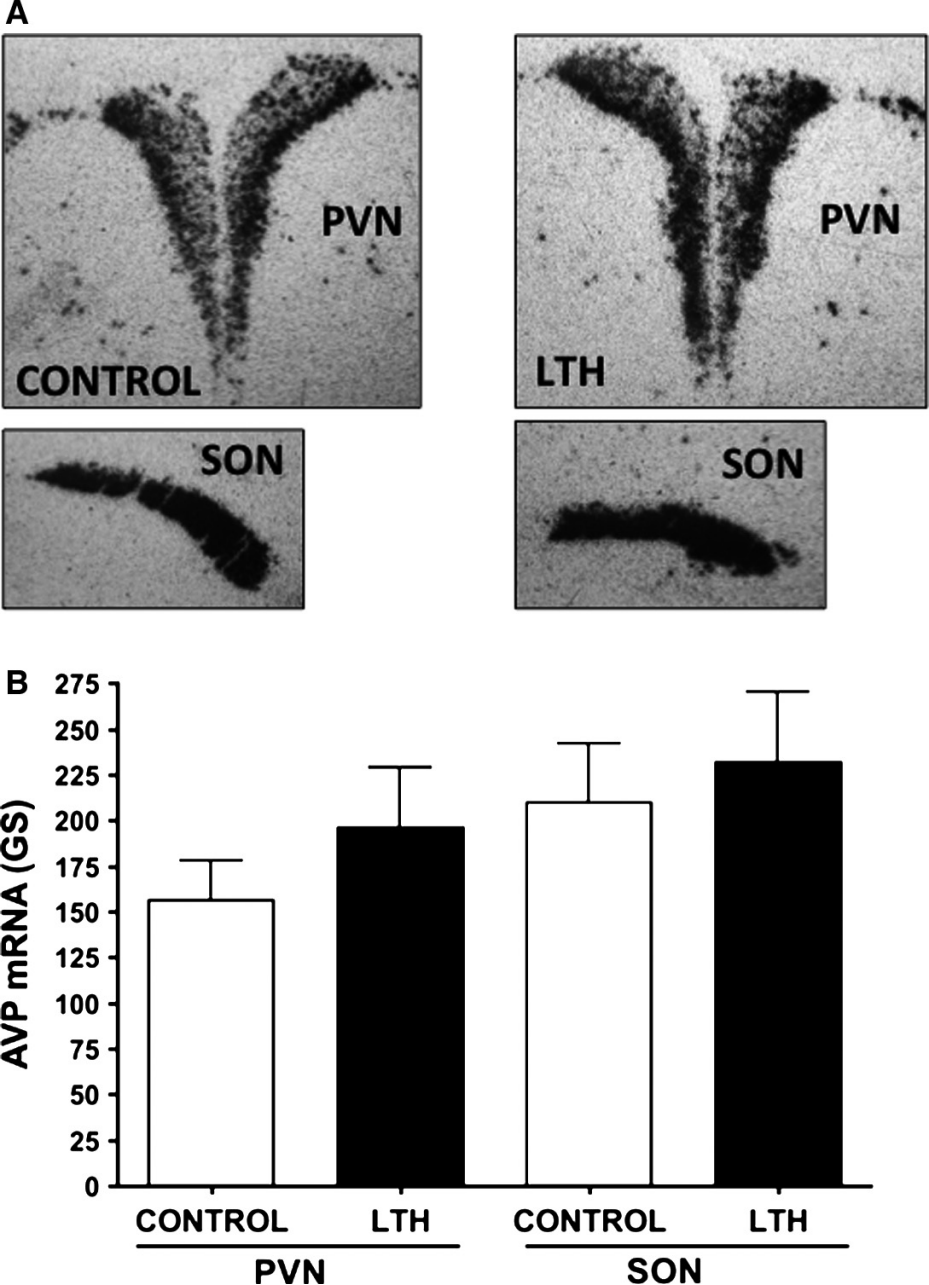
(A) Representative autoradiographs of AVP mRNA in the magnocellular paraventricular nucleus (PVN) and supraoptic nuclei (SON) of control and LTH fetal sheep. (B) AVP Messenger RNA hybridization signal strength (gray scale: GS) in PVN and SON from control and LTH fetal sheep. Data are means ± SEM from *n* = 5 fetuses per group.

Messenger RNA for CRH was not different in the mpPVN between LTH and control fetuses although there was a trend (*P* = 0.08) for CRH mRNA being elevated in the mpPVN of the LTH fetus (Fig. 2). However, the number of neurons with CRH hybridization signal per 1000 *μ*m^2^ of mpPVN was not different between LTH and control fetuses (LTH: 25 ± 6 vs. Control: 32 ± 9, mean ± SEM). Messenger RNA for AVP within parvocellular neurons of the mpPVN was significantly elevated (*P* < 0.05; Fig. 1) in LTH fetuses compared to control fetuses. In addition, we noted that the number of neurons with AVP hybridization signal within that of the parvocellular distribution was elevated per 1000 *μ*m^2^ of PVN imaged in LTH fetuses was greater compared to control (LTH: 29 ± 5 vs. Control: 11 ± 4, mean ± SEM). There were no differences in magnocellular AVP mRNA in PVN or SON between control and LTH fetuses (Fig. 3).

**Figure 4:**
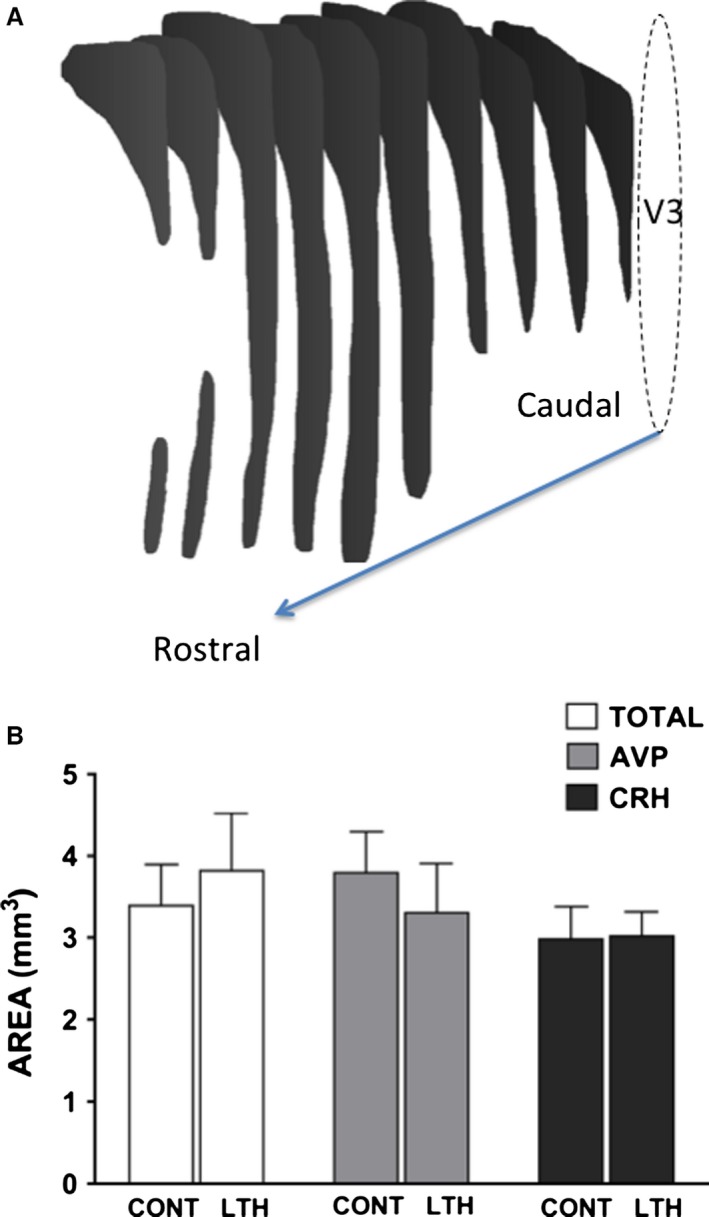
(A) Reconstruction of a unilateral ovine fetal PVN from the rostral to the caudal extent (bright‐field images of Nissl stained sections). These images were used to generate a stack and determine nuclei volume (V3 represents the third ventricle). (B) Effect of LTH on hypothalamic paraventricular nuclei volume and CRH and AVP hybridization volume in the hypothalamic paraventricular nuclei. Data are means ± SEM of area (mm^3^); *n* = 5 fetuses/group. Total volume was determined from Nissl stained sections through the entire length of the PVN. CRH and AVP hybridization was determined using dark‐field images of silver grains throughout the entire rostral‐caudal extent of the PVN.

The volumes for total PVN, AVP hybridizing area and CRH hybridizing area are presented in Figure 4. The volume of the total PVN obtained via bright‐field imaging of Nissl stained sections was not different between LTH and control fetuses (a rostral‐caudal rendering of stacked images of the ovine fetal PVN from Nissle stained sections is shown in Figure 4). Similarly, the hybridizing area for AVP (magno‐ and parvocellular divisions combined) was not different between LTH and control fetuses. The volume of the CRH hybridized region in the total rostral to caudal PVN (which included dorsal and dorsomedial parvocellular, periventricular parvocellular, and medial parvocellular subdivisions of the PVN) was also not different between the two groups (Fig. 4).

## Discussion

We previously described a significant impact of LTH on anterior pituitary corticotroph function by late gestation in fetal sheep consistent with LTH serving as a moderate stressor in these fetuses. This included increased processing of POMC to ACTH_1–39_ and increased basal plasma ACTH_1–39_ with a strong trend for elevated POMC in the LTH anterior pituitary (Myers et al. 2005). A potential hypothalamic regulation of the changes noted in anterior pituitary corticotropes was suggested by our previous findings of a downregulation of expression of the CRH_1_ receptor (CRHR_1_) coupled with an upregulation of the AVP V1b receptor in the LTH fetuses (Myers et al. 2005; Ducsay et al. 2009). LTH and normoxic control fetuses exhibited a similar release of ACTH_1–39_ in response to exogenous CRH, whereas LTH fetuses demonstrated an exaggerated ACTH_1–39_ release in response to exogenous AVP compared to the control fetuses (Ducsay et al. 2009). Since basal plasma cortisol concentrations are not different between LTH and control fetal sheep (Adachi et al. 2004; Imamura et al. 2004; Ducsay et al. 2009), these studies collectively supported a potential role for LTH modifying function of CRH and/or AVP neurons within the mpPVN leading to the noted changes in corticotrope function.

In this study, we found that LTH led to an increase in AVP mRNA hybridization signal within mpPVN neurons, whereas CRH mRNA in mpPVN neurons was not different between LTH and control fetuses, albeit there was a weak trend (*P* = 0.08) for CRH being elevated. The numbers of CRH hybridizing neurons within the mpPVN was similar between LTH and control fetuses as well. For AVP in the mpPVN, we noted an increased number of hybridizing neurons (number of neurons with a signal strength above background) as well as increase in hybridization signal strength per neuron (grains per area of mpPVN) in the LTH fetuses. This suggests either a recruitment of parvocellular neurons expressing AVP in response to LTH and/or an increase in AVP expression within CRH neurons in the mpPVN. We did not find an effect of LTH on the volume of the PVN (either total Nissl stained PVN or area of total PVN expression of CRH or AVP (magno‐ and parvocellular divisions) indicating that the effect of LTH on the PVN was on peptide expression and not volume in these nuclei. Our previously noted changes in the anterior pituitary of LTH fetal sheep suggested of a shift favoring AVP as the more potent ACTH secretagogue (increased V1b expression and response to exogenous AVP) (Myers et al. 2005; Ducsay et al. 2009). These data, coupled with the selective increase in mpPVN AVP expression, and lack of significant increase in CRH in the mpPVN of LTH fetuses support a switch to AVP as a primary ACTH secretagogue in response to the moderate sustained stressor of LTH.

The lack of increase in CRH mRNA in the mpPVN is not likely due to glucocorticoid negative feedback on this neuropeptide since basal cortisol levels are not elevated in the LTH fetus compared to control normoxic fetuses over the course of late gestation. Further, we also showed that there was no difference in hypothalamic glucocorticoid receptor number or affinity in fetuses exposed to LTH compared to normoxic controls (Adachi et al. 2004). However, a change in glucocorticoid feedback inhibition of the mpPVN at either the level of individual mpPVN neurons or at limbic structures such as hippocampus that provide negative feedback regulation of CRH, keeping this ACTH secretagogue from increasing in expression in the face of the chronic stress of LTH cannot be discounted at this time and will require further study.

In deference to our findings of an increase in AVP expression in parvocellular neurons within the PVN in response to LTH, we did not observe an effect of LTH on AVP mRNA levels in magnocellular neurons in either the PVN or SON. This indicates that the neuronal mechanisms mediating the increase in AVP expression in the population of mpPVN neurons is likely a direct effect of hypoxia per se but rather via hypoxia modulation of specific afferent input to parvocellular CRH/AVP neurons compared to the magnocellular neurons in the PVN and the SON. The magnocellular neurons project to the posterior pituitary and release AVP into the peripheral circulation where it functions primarily as an antidiuretic hormone and to a lesser degree, a pressor. As such, the magnocellular PVN and SON receive differential input from ascending control mechanisms that sense changes in plasma osmolality and blood pressure compared to the parvocellular neurons. It is, however, worth noting that Giussani et al., (Giussani et al. 1999) observed an ~7‐fold greater plasma AVP response in the high‐altitude adapted llama fetus compared to age matched fetal sheep in response to acute hypoxia. While we did not measure plasma AVP, it is possible that the LTH fetus, in response to a secondary stressor such as hypotension, hypovolemia or hypoxia, could elicit a greater release of AVP. Based on our findings however, this is not reflected by expression levels of this neuropeptide in the PVN and SON.

Earlier studies in adult sheep showed that the biosynthesis and secretion of ACTH is predominantly regulated by AVP and not CRH (Liu et al. 1994). However, unlike the adult, in the ovine fetus CRH and AVP both increase ACTH_1–39_ release in vitro and in vivo (Lu et al. 1994; Fora et al. 1996). During the final third of gestation in fetal sheep, Fora et al. (Fora et al. 1996) reported that ovine fetal anterior pituitary cells exhibit a progressive decline in ACTH_1–39_ release in response to CRH, while AVP‐induced ACTH release increases. This is supported by the findings of Lu et al., (1994) that CRH binding sites in the anterior pituitary declined during late gestation in the ovine fetus. Our prior results of an increase in V1b receptor expression coupled with decrease in CRH_1_ receptor expression are consistent with LTH advancing the normal ontogenic gain in AVP responsiveness of corticotropes noted during late gestation. The present findings of a increased AVP mRNA in the mpPVN suggest a similar adaptation at the level of the PVN favoring AVP as a major ACTH secretagogue in the LTH fetus. This may reflect the effect of chronic stress (LTH) on AVP expression since AVP gains prominence as an ACTH‐releasing factor in adult rats in response to chronic somatosensory stress (Aguilera et al. 2008). Chronic somatosensory stressors in rats also lead to an upregulation of anterior pituitary V1b receptors coupled with downregulation of anterior pituitary CRH_1_ receptors (Aguilera et al. 2008) similar to what we have reported in response to LTH in the fetal sheep (Myers et al. 2005). AVP appears to play a direct role in the decrease in CRH_1_ receptors in rodents in response to chronic stress as well (Hauger and Aguilera 1993). Thus, during chronic stress in both rats and in fetal sheep (LTH), the HPA axis may switch from CRH to AVP as an adaptive response (Ma et al. 1997; Aguilera and Rabadan‐Diehl 2000). Given the increased potency of AVP compared to CRH in ACTH release in the ovine fetus (Matthews and Challis 1995b, 1997), this response to LTH at the level of the PVN (enhanced AVP expression) may govern the adaptive changes we observed at the level of the anterior pituitary. Ultimately, as we have noted an enhanced cortisol response in the LTH fetus to a secondary stressor (Adachi et al. 2004; Imamura et al. 2004), the adaptive changes at the level of the PVN and anterior pituitary may provide a mechanism via which these fetuses exhibit an enhanced adrenocortical stress response to a secondary acute stress.

There are dramatic changes rendered at the level of both the mpPVN and the anterior pituitary corticotroph by development under conditions of LTH that result in an apparent enhancement of function of the HPA axis, including increased basal ACTH_1–39_ and enhanced release of ACTH_1–39_ in response to a superimposed acute stressor. Despite these alterations, the LTH fetus exhibits a normal ontogenic rise in fetal plasma cortisol. As we have previously reported, this is achieved largely at the level of the adrenal cortex via decreased expression of key steroidogenic enzymes (CYP11A1 and CYP17) and decreased ACTH receptor expression. This limits the capacity for the adrenocortical cells to respond to the elevated basal ACTH. As we have shown this is achieved by both intra‐adrenal mechanisms (nitric oxide) that limit ACTH signaling, and extra‐adrenal mechanisms, namely leptin, that reduce CYP11A1 and CYP17 expression. However, the changes observed at the level of the anterior pituitary and in this study at the PVN, contribute significantly toward these fetuses being able to mount an enhanced acute cortisol response as a defense mechanism.

An additional adrenocortical/neural mechanism that may come into play in the adaptation observed in response to LTH is a reflex arc initiated via carotid chemoreceptors through activation of splanchnic nerve efferents supplying the adrenal gland. Myers and colleagues (Myers et al. 1990) found that severing splanchnic innervation to the ovine fetal adrenal glands during late gestation attenuated the cortisol response to acute hypotension while Giussani and co‐workers (Giussani et al. 1994) reported that carotid sinus nerve section in the late gestation sheep fetus delayed the rise in plasma cortisol in response to acute hypoxia. The effect could largely be attributed to changes in adrenocortical sensitivity to ACTH and that carotid chemo and baroreceptor mechanisms may provide both an afferent (central) and efferent (splanchnic) means of integrating the acute cortisol response without need to dramatically alter the anterior pituitary ACTH response.

As we have discussed above, and by Riquelme et al., (1998), limiting the adrenocortical response to ACTH may be a physiologic adaption to prolonged periods of hypoxia in utero. The LTH ovine fetus may invoke similar mechanism as high‐altitude adapted species such as llama (Riquelme et al. 1998, 2002). Of particular interest in light of our prior studies showing the involvement of NO signaling in the adaptive response in the LTH fetal sheep, these authors (Riquelme et al. 2002) demonstrated that NO plays an important role in the adaptive responses of the fetal llama adrenal cortex to its high‐altitude environment. In this study, we focused on the adaptation at the level of the hypothalamic PVN to LTH. However, in addition to regulation anterior pituitary corticotroph function via mpPVN CRH and AVP, divisions of the PVN such as the dorsomedial PVN (dm) send projections to brainstem nuclei controlling autonomic function such as periaqueductal and pontine gray as well as the nucleus of the solitary tract (Thompson et al. 1996). As such, LTH, via the dmPVN could alter brainstem outflow affecting function of the splanchnic nerve. However, our observation that sino‐aortic denervation did not significantly alter the cortisol response to a secondary stressor in the late gestation LTH fetus (Kato et al. 2003) would support that the adaptation of the HPA axis resulting in the noted changes in adrenocortical function are largely governed by classic endocrine systems (e.g., PVN, ACTH, leptin) as well as intra‐adrenal mechanisms (NO). Thus, these fetuses reveal a remarkable adaptation to development under conditions of sustained moderate physiologic stress.

## Conflict of Interest

None declared.
